# Biology and applications of endophytic insect-pathogenic fungi

**DOI:** 10.1371/journal.ppat.1007831

**Published:** 2019-07-18

**Authors:** Margaret Branine, Anna Bazzicalupo, Sara Branco

**Affiliations:** Department of Microbiology and Immunology, Montana State University, Bozeman, Montana, United States of America; Geisel School of Medicine at Dartmouth, UNITED STATES

## Overview

Endophytic insect-pathogenic fungi (EIPF) are both plant mutualists and insect pathogens, living inside plant tissues without causing any symptoms to their plant partner while also parasitizing and killing insects [1]. These interactions can occur simultaneously and lead to the demise of plant insect pests. The ecology and evolution of EIPF are still not fully understood; however, several studies have investigated their diversity [2,3], the mechanisms of plant and insect infection [4,5], and the nature of plant–insect–fungus relationships [6,7]. These multikingdom interactions are of particular interest not only because EIPF are an ideal model for understanding the mechanisms of symbioses, but they are also widely used for practical applications and particularly relevant to agricultural pest control [8]. Here, we provide an overview on EIPF by reviewing what is currently known about their evolution, ecology, and mechanisms of insect and plant colonization. We also address real-world applications of EIPF and identify possible research directions for the field in the future.

## The EIPF lifestyle evolved in the fungal order Hypocreales (Ascomycota)

Insect pathogenicity is a relatively common nutritional mode among fungi and has evolved independently multiple times within different lineages [9,10]. However, most insect-pathogenic fungi do not have the ability to establish themselves in living plant tissues. EIPF evolved in the order Hypocreales (Ascomycota) [2,3], with the generalist insect pathogens *Beauveria* and *Metarhizium* (in the families Cordycipitaceae and Clavicipitaceae, respectively) as the most well-studied EIPF genera. The evolution of EIPF is currently not fully understood; however, a study investigating divergence of the genes involved in insect and plant associations suggested *Metarhizium*’s association with plants was more likely to have driven diversification than insect pathogenicity [11]. This genus displays a large number of genes specific to plant degradation that allow the digestion of plant material, suggesting it may have evolved from fungi associated with plants [12]. Ancestral character reconstruction based on broad phylogenetic sampling of the Hypocreales suggests that the order’s ancestral ecology also involved insect pathogenicity but recovered *Metarhizium* or *Beauveria* as related to plant-associated clades [2,3]. The limited breadth and depth of sampling throughout the Hypocreales and assigning a single lifestyle to each species (endophytic, plant pathogen, or insect pathogen) may have limited the insight of these results. More intense sampling and comprehensive scoring of species’ endophytic habits and insect pathogenicity abilities would assist in detecting further EIPF lineages and widen our understanding of both their distribution across the fungal tree of life and their evolutionary history. For example, linking environmental surveys of endophytic fungal diversity with phylogenetic studies could be a powerful approach for detecting more EIPF. Given the high diversity of fungal insect pathogens, it is possible that many more are also endophytic and have so far been overlooked as EIPF.

## The ecology of EIPF involves intimate multikingdom nutrient transactions

**Fig 1:**
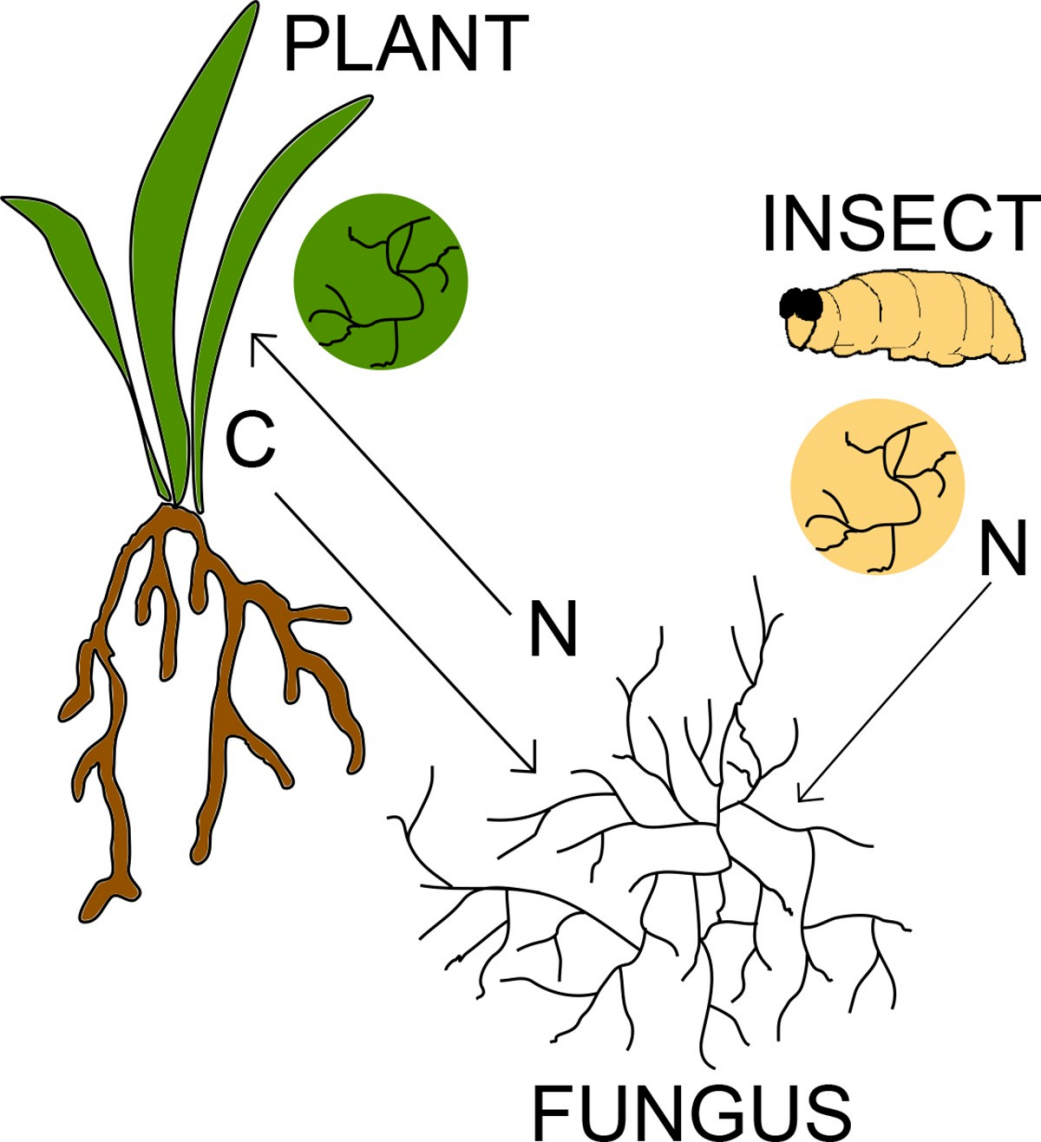
Model of fungal-mediated nutrient transfer in tripartite interactions between EIPF, plants, and insects. The fungal hyphae colonize both partners (circles depict plant and larval tissue colonized by EIPF mycelium) with different effects. The fungus obtains nitrogen (N) by digesting insect tissue and transfers it to the plant. In addition, plant-derived carbon (C) is moved from the plant to the fungus. EIPF, endophytic insect-pathogenic fungi.

EIPF establish mutualistic associations with plants and parasitize insects. These associations can occur simultaneously, with one single fungal individual colonizing plant tissues and infecting insects, forming a tripartite interaction and allowing for nutrient transfer across the fungus, the plant, and the insect (Fig 1). Most of our understanding of these interactions comes from studies investigating *Metarhizium robertsii*, whose mycelium colonizes both plant root cells and the soil larvae feeding on root tissue. Elegant experiments using radioactive isotopes showed *M*. *robertsii* both receiving carbon from the plant partner [7] and transferring nitrogen from insects to plant roots [6]. These microcosm experiments tracked ^15^N and ^13^C in *M*. *robertsii*, plants, and larvae, demonstrating that insect-derived nitrogen is moved to the plant only when the fungus is present and that plant-derived carbon is transferred to the fungus and incorporated in fungal carbohydrates such as chitin and trehalose.

These experiments clearly show a complex tripartite interaction, characterized by transfer of nutrients across EIPF, plants, and insects. Although these studies provide a powerful insight on mediated nutrient transfer, it is still unknown whether or how these mechanisms can be generalized and applied to fungi other than *M*. *robertsii*. Future research collecting experimental evidence on diverse EIPF, plant, and insect species will contribute to quantify the importance of nutrient transfer for each party’s survival and test the stability of these interactions over time. Such experiments will shed light on how widespread these three-way symbioses are and how they operate in nature.

## EIPF have similar mechanisms to infect both plant and insect hosts

In order for EIPFs to be successful symbionts of plants and insects, they need to be able to invade and establish in both organisms. The insect infection processes in the genera *Beauveria* and *Metarhizium* have been well studied and serve as a general model for EIPF [1,13]. These fungi seem to use similar mechanisms to penetrate and establish inside their plant and insect hosts, with similar genes involved in insect infection and establishment in plants. Such genes have been hypothesized to derive from gene duplications [5] or horizontal gene transfer [14,15], implying shared processes in becoming plant and insect symbionts. We currently know more about the genes involved in EIPF insect infection than the genes involved in plant colonization [1].

Fungal insect colonization starts with the adhesion of asexual spores to the host surface, followed by penetration into the living tissue and proliferation inside the body cavity [16]. In order to successfully parasitize insects, the fungus needs to evade the animal’s immune system. Once established, the fungus kills the insect in a rapid process. In *M*. *robertsii*, the adhesin MAD1 is essential for insect cuticle conidial spore adhesion [5], whereas hydrophobins play this role in *Beauveria* [17]. Surface proteins on the conidia recognize insect-specific compounds and initiate degradation of the insect cuticle. Following successful adherence, the conidia germinate to form hyphae. Degradation of the cuticle is accomplished via enzymatic activity by various proteases [18] and the mechanical pressure exerted by specialized infection hyphal structures, such as appressoria [19]. Once the fungus penetrates the cuticle, it reaches the insect hemolymph, where it differentiates into blastospores (yeast-like asexual spores). Blastospores absorb nutrients in the hemocoel and produce insecticidal metabolites, such as beauvericin [20] and destruxins [21], resulting in insect death within a matter of days. These insect pathogens can also produce antimicrobial compounds post-insect death that prevent microbial competition and assure nutrients are fully available for fungal growth and reproduction [22].

EIPF also colonize plants, establishing as mutualists in their tissues. Different EIPF preferentially establish in distinct plant parts, with *Metarhizium* primarily found in roots and *Beauveria* within multiple plant tissues [23]. The plant adhesion process in *M*. *robertsii* depends on the adhesin MAD2, a very similar protein to the MAD1 adhesin involved in insect attachment [5]. As in insect cuticle penetration, proteases degrade the plant cell wall to allow EIPF access to plant tissues [19]. In order to establish as an endophyte, the fungus avoids plant defenses in a process thought to be mediated by diffusible communication molecules, as in other plant-mutualistic fungi not associated with insects [19]. In contrast to the antagonistic interaction with insects, EIPF establish as plant mutualists and provide nitrogen to the plant [6], promote plant growth and productivity [24], and combat plant pests [1].

Details on all the genes involved in EIPF plant establishment, as well as on fungal-plant communication, are not well known. However, research on *M*. *robertsii* has shed light on plant colonization and communication, unveiling some of the mechanisms involved in endophytism. Plant establishment in this fungus is dependent on a raffinose transporter and an extracellular invertase involved in sucrose hydrolysis [22,23]. Raffinose and sucrose are abundant in root exudates, and these molecules are essential for *M*. *robertsii* growth in the rhizosphere and for root competence. This species is well known for promoting plant growth and plant benefits correlate with fungal association to roots [24,25,26]. *M*. *robertsii* promotes root growth through an auxin-dependent mechanism that might also be linked to insect pathogenicity, as auxins have been shown to enhance insect virulence [27,28]. Future research focusing on a broad sample of EIPF lineages will unveil more genes involved in interactions with both insects and plants, inform on the existence of distinct mechanisms across different fungal clades, and contribute to a more general understanding of symbiotic interactions with plants and insects.

## EIPF as tools for agricultural and biotechnological applications

The potential for EIPF in practical applications has been explored since the discovery of *Beauveria bassiana* in the early nineteenth century [29]. These fungi are well known for promoting plant growth and enhancing insect virulence [27,28,30,31,32] and are currently explored for pest control in agriculture [33]. EIPF are also exploited for their secondary metabolites, which are useful in biotechnology and medicine.

Endophytic fungi are currently used as tools to improve crop productivity [34], and EIPF in particular have the added benefit of also acting as pest-control agents. It is clear that EIPF improve plant growth and crop yield and are effective at protecting plants against insect attacks both in the lab and the field [27,28,30,31,32,35]. In fact, biocontrol of invertebrate pests is an attractive alternative to the current prolific use of synthetic pesticides, which have detrimental environmental impacts [36]. Application of microbial biopesticides provides a potential alternative that is comparably environmentally sound, and effective EIPF isolates are currently commercially available for invertebrate pest control, including species of *Beauveria* and *Metarhizium* [37,38]. For example, *B*. *bassiana* alone is effective for controlling herbivorous insects in maize [35], cotton [39], banana [40], white jute [41], and poppy [42]. This species also establishes in wheat plants, controlling for cotton leafworm larvae (a widespread pest of many cultivated crops) while also increasing spike production [32]. EIPF may also help control plant disease, making them promising for biocontrol of other pathogenic agents [43]. Recent efforts have focused on improving fungal virulence against their insect hosts to make EIPF more efficient [1]. While the use of EIPF in agriculture has advanced the field of biocontrol and these fungi hold great promise for improving food production worldwide, their application also warrants caution. Many of these fungi are insect generalists [1] and may affect species other than the target pest, resulting in unwanted consequences to the local ecology. For example, *B*. *bassiana* conidia are known to effectively kill *Amblyseius swirskii*, a predatory mite that feeds on insects and other mites that is also used for biocontrol in agriculture [44]. It is therefore crucial to perform adequate tests before field EIPF applications as to assure off-target infections are not detrimental to crops and local ecosystems.

EIPF are most notorious for applications in agriculture, but their study has also spawned use in the medical sciences. Many EIPF secondary metabolites have antimicrobial and cytotoxic activities [45], such as beauvericin, oosporein, and taxol. It is thought that EIPF synthesize such compounds in order to kill insects as well as to limit bacterial competition within the host [22]. Because of their cytotoxic properties, these molecules have been investigated for anticancer therapy. Beauvericin has been shown to slow the migration of prostate and breast cancer cells [46], making it a promising candidate for anticancer treatments. Fungal-derived taxol is also known for being effective in inducing apoptosis and preventing tumor proliferation in human cancer cells [47,48], and current efforts are being made to obtain high taxol yields from fungal cultures, including from species in the genus *Metarhizium* [49]. Further research investigating the potential of diverse EIPF lineages for agricultural biocontrol as well as the discovery of novel fungal secondary metabolites and their activities will certainly lead to new innovations and applications.

In conclusion, the study of EIPF systems has great potential to elucidate fundamental questions on the ecology and evolution of multispecies interactions and to provide solutions to agricultural and medical problems.

## References

[1] Moonjely S, Barelli L, Bidochka MJ. Insect Pathogenic Fungi as Endophytes In: St Leger RJ, editor. Advances in Genetics. Academic Press; 2016 pp. 107–135. 10.1016/bs.adgen.2015.12.004 27131324

[2] Spatafora JW, Sung G-H, Sung J-M, Hywel-Jones NL, White JF. Phylogenetic evidence for an animal pathogen origin of ergot and the grass endophytes. Mol Ecol. 2007;16: 1701–1711. 10.1111/j.1365-294X.2007.03225.x 17402984

[3] Zhang W, Zhang X, Li K, Wang C, Cai L, Zhuang W, et al Introgression and gene family contraction drive the evolution of lifestyle and host shifts of hypocrealean fungi. Mycology. 2018;9: 176–188. 10.1080/21501203.2018.1478333 30181924 PMC6115877

[4] Wang C, St Leger RJ. A collagenous protective coat enables *Metarhizium anisopliae* to evade insect immune responses. Proc Natl Acad Sci USA. 2006;103: 6647–6652. 10.1073/pnas.0601951103 16614065 PMC1458935

[5] Wang C, St Leger RJ. The MAD1 adhesin of *Metarhizium anisopliae* links adhesion with blastospore production and virulence to insects, and the MAD2 adhesin enables attachment to plants. Eukaryotic Cell. 2007;6: 808–816. 10.1128/EC.00409-06 17337634 PMC1899246

[6] Behie SW, Zelisko PM, Bidochka MJ. Endophytic Insect-Parasitic Fungi Translocate Nitrogen Directly from Insects to Plants. Science. 2012;336: 1576–1577. 10.1126/science.1222289 22723421

[7] Behie SW, Moreira CC, Sementchoukova I, Barelli L, Zelisko PM, Bidochka MJ. Carbon translocation from a plant to an insect-pathogenic endophytic fungus. Nat Commun. 2017;8: 14245 10.1038/ncomms14245 28098142 PMC5253661

[8] Vega FE, Goettel MS, Blackwell M, Chandler D, Jackson MA, Keller S, et al Fungal entomopathogens: new insights on their ecology. Fungal Ecology. 2009;2: 149–159. 10.1016/j.funeco.2009.05.001

[9] Zheng P, Xia Y, Zhang S, Wang C. Genetics of *Cordyceps* and related fungi. Appl Microbiol Biotechnol. 2013;97: 2797–2804. 10.1007/s00253-013-4771-7 23435902

[10] Haelewaters D, Page RA, Pfister DH. Laboulbeniales hyperparasites (Fungi, Ascomycota) of bat flies: Independent origins and host associations. Ecology and Evolution. 2018;8: 8396–8418. 10.1002/ece3.4359 30250711 PMC6145224

[11] Wyrebek M, Bidochka MJ. Variability in the Insect and Plant Adhesins, Mad1 and Mad2, within the Fungal Genus *Metarhizium* Suggest Plant Adaptation as an Evolutionary Force. PLoS ONE. 2013;8: e59357 10.1371/journal.pone.0059357 23516629 PMC3596358

[12] Gao Q, Jin K, Ying S-H, Zhang Y, Xiao G, Shang Y, et al Genome Sequencing and Comparative Transcriptomics of the Model Entomopathogenic Fungi *Metarhizium anisopliae* and *M*. *acridum*. PLoS Genet. 2011;7: e1001264 10.1371/journal.pgen.1001264 21253567 PMC3017113

[13] Valero-Jiménez CA, Wiegers H, Zwaan BJ, Koenraadt CJM, van Kan JAL. Genes involved in virulence of the entomopathogenic fungus *Beauveria bassiana*. Journal of Invertebrate Pathology. 2016;133: 41–49. 10.1016/j.jip.2015.11.011 26628209

[14] Screen SE, St Leger RJS. Cloning, Expression, and Substrate Specificity of a Fungal Chymotrypsin evidence for lateral gene transfer from an actinomycete bacterium. J Biol Chem. 2000;275: 6689–6694. 10.1074/jbc.275.9.6689 10692479

[15] Zhang Q, Chen X, Xu C, Zhao H, Zhang X, Zeng G, et al Horizontal gene transfer allowed the emergence of broad host range entomopathogens. PNAS. 2019;116: 7982–7989. 10.1073/pnas.1816430116 30948646 PMC6475382

[16] Vega FE, Meyling NV, Luangsa-ard JJ, Blackwell M. Fungal Entomopathogens In: Vega FE, Kaya HK, editors. Insect Pathology (Second Edition). San Diego: Academic Press; 2012 pp. 171–220. 10.1016/B978-0-12-384984-7.00006-3

[17] Zhang S, Xia YX, Kim B, Keyhani NO. Two hydrophobins are involved in fungal spore coat rodlet layer assembly and each play distinct roles in surface interactions, development and pathogenesis in the entomopathogenic fungus, *Beauveria bassiana*. Molecular Microbiology. 2011;80: 811–826. 10.1111/j.1365-2958.2011.07613.x 21375591

[18] St. Leger RJ. The role of cuticle-degrading proteases in fungal pathogenesis of insects. Can J Bot. 1995;73: 1119–1125. 10.1139/b95-367

[19] Barelli L, Moonjely S, Behie SW, Bidochka MJ. Fungi with multifunctional lifestyles: endophytic insect pathogenic fungi. Plant Mol Biol. 2016;90: 657–664. 10.1007/s11103-015-0413-z 26644135

[20] Xu Y, Orozco R, Wijeratne EMK, Gunatilaka AAL, Stock SP, Molnár I. Biosynthesis of the cyclooligomer depsipeptide beauvericin, a virulence factor of the entomopathogenic fungus *Beauveria bassiana*. Chem Biol. 2008;15: 898–907. 10.1016/j.chembiol.2008.07.011 18804027

[21] Wahlman M, Davidson BS. New Destruxins from the Entomopathogenic Fungus *Metarhizium anisopliae*. J Nat Prod. 1993;56: 643–647. 10.1021/np50094a034

[22] Fan Y, Liu X, Keyhani NO, Tang G, Pei Y, Zhang W, et al Regulatory cascade and biological activity of *Beauveria bassiana* oosporein that limits bacterial growth after host death. Proc Natl Acad Sci USA. 2017;114: E1578–E1586. 10.1073/pnas.1616543114 28193896 PMC5338512

[23] Behie SW, Jones SJ, Bidochka MJ. Plant tissue localization of the endophytic insect pathogenic fungi *Metarhizium* and *Beauveria*. Fungal Ecology. 2015;13: 112–119. 10.1016/j.funeco.2014.08.001

[24] Behie SW, Bidochka MJ. Ubiquity of Insect-Derived Nitrogen Transfer to Plants by Endophytic Insect-Pathogenic Fungi: an Additional Branch of the Soil Nitrogen Cycle. Appl Environ Microbiol. 2014;80: 1553–1560. 10.1128/AEM.03338-13 24334669 PMC3957595

[25] Fang W, St Leger RJS. Mrt, a Gene Unique to Fungi, Encodes an Oligosaccharide Transporter and Facilitates Rhizosphere Competency in *Metarhizium robertsii*. Plant Physiology. 2010;154: 1549–1557. 10.1104/pp.110.163014 20837701 PMC2971628

[26] Liao X, Fang W, Lin L, Lu H-L, St Leger RJS. *Metarhizium robertsii* Produces an Extracellular Invertase (MrINV) That Plays a Pivotal Role in Rhizospheric Interactions and Root Colonization. PLoS ONE. 2013;8: e78118 10.1371/journal.pone.0078118 24205119 PMC3804458

[27] Sasan RK, Bidochka MJ. The insect-pathogenic fungus *Metarhizium robertsii* (Clavicipitaceae) is also an endophyte that stimulates plant root development. American Journal of Botany. 2012;99: 101–107. 10.3732/ajb.1100136 22174335

[28] Liao X, O’Brien TR, Fang W, St Leger RJ. The plant beneficial effects of *Metarhizium* species correlate with their association with roots. Appl Microbiol Biotechnol. 2014;98: 7089–7096. 10.1007/s00253-014-5788-2 24805846

[29] Ortiz-Urquiza A, Keyhani NO. Action on the Surface: Entomopathogenic Fungi versus the Insect Cuticle. Insects. 2013;4: 357–374. 10.3390/insects4030357 26462424 PMC4553469

[30] Liao X, Lovett B, Fang W, St Leger RJ. *Metarhizium robertsii* produces indole-3-acetic acid, which promotes root growth in Arabidopsis and enhances virulence to insects. Microbiology. 2017;163: 980–991. 10.1099/mic.0.000494 28708056

[31] Raya–Díaz S, Quesada–Moraga E, Barrón V, del Campillo MC, Sánchez–Rodríguez AR. Redefining the dose of the entomopathogenic fungus *Metarhizium brunneum* (Ascomycota, Hypocreales) to increase Fe bioavailability and promote plant growth in calcareous and sandy soils. Plant Soil. 2017;418: 387–404. 10.1007/s11104-017-3303-0

[32] Sánchez-Rodríguez AR, Raya-Díaz S, Zamarreño ÁM, García-Mina JM, del Campillo MC, Quesada-Moraga E. An endophytic *Beauveria bassiana* strain increases spike production in bread and durum wheat plants and effectively controls cotton leafworm (*Spodoptera littoralis*) larvae. Biological Control. 2018;116: 90–102. 10.1016/j.biocontrol.2017.01.012

[33] Mascarin GM, Jaronski ST. The production and uses of *Beauveria bassiana* as a microbial insecticide. World J Microbiol Biotechnol. 2016;32: 177 10.1007/s11274-016-2131-3 27628337

[34] Redman RS, Rodriguez RJ. The Symbiogenic Tango: Achieving Climate-Resilient Crops Via Mutualistic Plant-Fungal Relationships In: Doty SL, editor. Functional Importance of the Plant Microbiome: Implications for Agriculture, Forestry and Bioenergy. Cham: Springer International Publishing; 2017 pp. 71–87. 10.1007/978-3-319-65897-1_5

[35] Kabaluk JT, Ericsson JD. *Metarhizium anisopliae* Seed Treatment Increases Yield of Field Corn When Applied for Wireworm Control. Agronomy Journal. 2007;99: 1377–1381. 10.2134/agronj2007.0017N

[36] Glare T, Caradus J, Gelernter W, Jackson T, Keyhani N, Köhl J, et al Have biopesticides come of age? Trends Biotechnol. 2012;30: 250–258. 10.1016/j.tibtech.2012.01.003 22336383

[37] Castrillo LA, Griggs MH, Ranger CM, Reding ME, Vandenberg JD. Virulence of commercial strains of *Beauveria bassiana* and *Metarhizium brunneum* (Ascomycota: Hypocreales) against adult *Xylosandrus germanus* (Coleoptera: Curculionidae) and impact on brood. Biological Control. 2011;58: 121–126. 10.1016/j.biocontrol.2011.04.010

[38] Bing LA, Lewis LC. Suppression of *Ostrinia nubilalis* (Hübner) (Lepidoptera: Pyralidae) by Endophytic *Beauveria bassiana* (Balsamo) Vuillemin. Environ Entomol. 1991;20: 1207–1211. 10.1093/ee/20.4.1207

[39] Lopez DC, Zhu-Salzman K, Ek-Ramos MJ, Sword GA. The Entomopathogenic Fungal Endophytes *Purpureocillium lilacinum* (Formerly Paecilomyces lilacinus) and *Beauveria bassiana* Negatively Affect Cotton Aphid Reproduction under Both Greenhouse and Field Conditions. PLoS ONE. 2014;9: e103891 10.1371/journal.pone.0103891 25093505 PMC4122372

[40] Akello J, Dubois T, Coyne D, Kyamanywa S. Endophytic *Beauveria bassiana* in banana (*Musa* spp.) reduces banana weevil (*Cosmopolites sordidus*) fitness and damage. Crop Protection. 2008;27: 1437–1441. 10.1016/j.cropro.2008.07.003

[41] Biswas C, Dey P, Satpathy S, Satya P, Mahapatra BS. Endophytic colonization of white jute (*Corchorus capsularis*) plants by different *Beauveria bassiana* strains for managing stem weevil (*Apion corchori*). Phytoparasitica. 2013;41: 17–21. 10.1007/s12600-012-0257-x

[42] Quesada-Moraga E, Muñoz-Ledesma FJ, Santiago-Álvarez C. Systemic Protection of Papaver somniferum L. Against *Iraella luteipes* (Hymenoptera: Cynipidae) by an Endophytic Strain of *Beauveria bassiana* (Ascomycota: Hypocreales). Environ Entomol. 2009;38: 723–730. 10.1603/022.038.0324 19508781

[43] Jaber LR, Ownley BH. Can we use entomopathogenic fungi as endophytes for dual biological control of insect pests and plant pathogens? Biological Control. 2018;116: 36–45. 10.1016/j.biocontrol.2017.01.018

[44] Seiedy M, Tork M, Deyhim F. Effect of the entomopathogenic fungus *Beauveria bassiana* on the Predatory Mite *Amblyseius swirskii* (Acari: Phytoseiidae) as a Non-Target Organism. Syst Appl Acarol. 2015;20: 241–250. 10.11158/saa.20.3.2

[45] Donzelli BGG, Krasnoff SB. Molecular Genetics of Secondary Chemistry in *Metarhizium* Fungi In: St Leger RL, editor. Advances in Genetics. Academic Press; 2016 pp. 365–436. 10.1016/bs.adgen.2016.01.005 27131330

[46] Wu Q, Patocka J, Nepovimova E, Kuca K. A Review on the Synthesis and Bioactivity Aspects of Beauvericin, a *Fusarium* Mycotoxin. Front Pharmacol. 2018;9 10.3389/fphar.2018.01338 30515098 PMC6256083

[47] Wang X, Wang C, Sun Y-T, Sun C-Z, Zhang Y, Wang X-H, et al Taxol produced from endophytic fungi induces apoptosis in human breast, cervical and ovarian cancer cells. Asian Pac J Cancer Prev. 2015;16: 125–131. 10.7314/apjcp.2015.16.1.125 25640339

[48] Gokul Raj K, Manikandan R, Arulvasu C, Pandi M. Anti-proliferative effect of fungal taxol extracted from *Cladosporium oxysporum* against human pathogenic bacteria and human colon cancer cell line HCT 15. Spectrochimica Acta Part A: Molecular and Biomolecular Spectroscopy. 2015;138: 667–674. 10.1016/j.saa.2014.11.036 25544183

[49] EL-Maali NA, Mohrram AM, El-Kashef H, Gamal K. Novel resources of Taxol from endophytic and entomopathogenic fungi: Isolation, characterization and LC-Triple mass spectrometric quantification. Talanta. 2018;190: 466–474. 10.1016/j.talanta.2018.07.089 30172534

